# Investigation of novel metabolites potentially involved in the pathogenesis of coronary heart disease using a UHPLC-QTOF/MS-based metabolomics approach

**DOI:** 10.1038/s41598-017-15737-3

**Published:** 2017-11-10

**Authors:** Yiping Li, Dong Zhang, Yuan He, Changzhe Chen, Chenxi Song, Yanyan Zhao, Yinxiao Bai, Yang Wang, Jielin Pu, Jingzhou Chen, Yuejin Yang, Kefei Dou

**Affiliations:** State Key Laboratory of Cardiovascular Disease, Fuwai Hospital, National Centre for Cardiovascular Diseases, Chinese Academy of Medical Sciences & Peking Union Medical College, Beijing, 100037 People’s Republic of China

## Abstract

Coronary heart disease (CHD) is associated with complex metabolic disorders, but its molecular aetiology remains unclear. Using a novel nontargeted metabolomics approach, we explored the global metabolic perturbation profile for CHD. Blood samples from 150 patients with severe obstructive CHD and 150 angiographically normal controls were collected. Metabolic fingerprinting was performed by ultra-high performance liquid chromatography coupled to quadruple time-of-flight mass spectrometry (UHPLC-QTOF/MS) technique. After adjusting for CHD traditional risk factors and metabolic batch, a comprehensive list of 105 metabolites was found to be significantly altered in CHD patients. Among the metabolites identified, six metabolites were discovered to have the strongest correlation with CHD after adjusting for multiple testing: palmitic acid (β = 0.205; p < 0.0001), linoleic acid (β = 0.133; p < 0.0001), 4-pyridoxic acid (β = 0.142; p < 0.0001), phosphatidylglycerol (20:3/2:0) (β = 0.287; p < 0.0001), carnitine (14:1) (β = 0.332; p < 0.0001) and lithocholic acid (β = 0.224; p < 0.0001); of these, 4-pyridoxic acid, lithocholic acid and phosphatidylglycerol (20:3/2:0) were, to the best of our knowledge, first reported in this study. A logistic regression model further quantified their positive independent correlations with CHD. In conclusion, this study surveyed a broad panel of nontargeted metabolites in Chinese CHD populations and identified novel metabolites that are potentially involved in CHD pathogenesis.

## Introduction

Coronary heart disease (CHD) is one of the leading causes of mortality worldwide, but its pathogenic mechanism remains poorly understood. It is well-known that CHD is linked with complex metabolic disorders, such as obesity, insulin resistance, and diabetes^1,2^. And certain circulating metabolites, such as homocysteine, cholesterol and triglycerides, have been recognized for decades as being strongly associated with CHD^3–5^. Beyond these well-known associations, researchers believe that there might still be many other dysregulated metabolites that have yet to be described. Therefore, metabolomics, a powerful approach to detect a broad spectrum of small-molecule metabolites, is increasingly being applied to CHD studies and is leading to novel discoveries of metabolic biomarkers and implications of their causal relationship to CHD pathogenesis^6–8^. Previous metabolomics studies concerning CHD, with few exceptions, were assessed on targeted metabolomics platforms^6,7,9–11^. These studies examined a predefined group of potential metabolites that was mostly culled from diabetes metabolomics studies^6,7,9,10^. Meanwhile, the nontargeted metabolomics approach provides a global fingerprint of information through the simultaneous measurement of as many metabolites as possible and may better contribute to discovery-driven research on coronary atheroma^8,12^. Our study used an ultra-high performance liquid chromatography coupled with the quadruple time-of-flight mass spectrometry (UHPLC-QTOF/MS)-based nontargeted metabolomics approach to explore the molecular perturbation profile of severe CHD patients compared with angiographically normal controls. We aimed to extend the current knowledge beyond previously reported targeted metabolite changes by examining the global plasma metabolomics profile of coronary heart disease and searching for new metabolites that are potentially involved in CHD pathogenesis.

## Methods

### Participant recruitment

**Figure 1:**
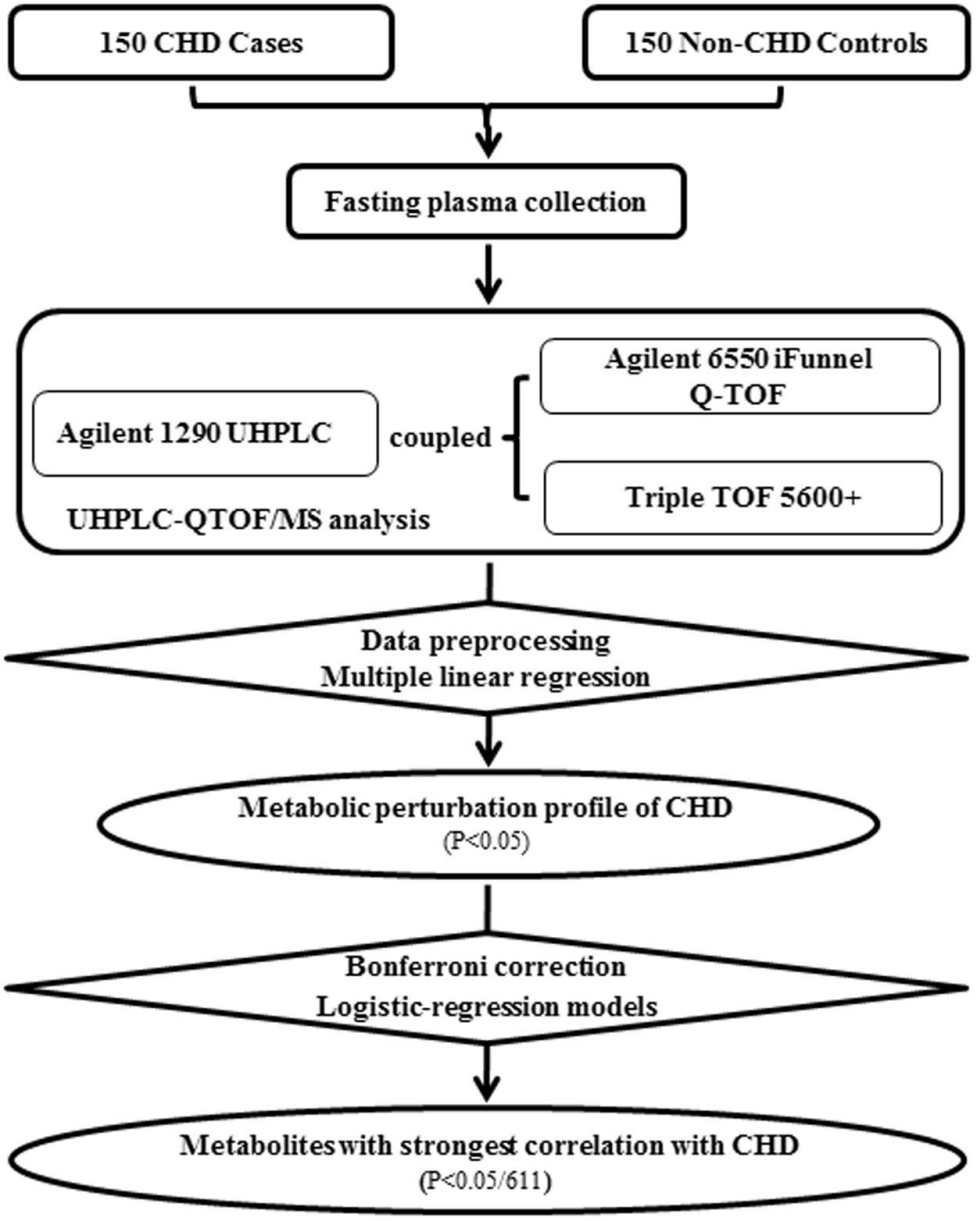
Flowchart of study strategy.

Blood samples of 980 patients who underwent coronary angiography to evaluate CHD at FuWai Hospital between June 2011 and March 2015 were collected for our study. We excluded participants who had organic heart diseases (rheumatic heart disease, etc.), liver/renal dysfunctions, a severe infection or malignant tumours and hyperthyroidism or any other autoimmune disease, resulting in a cohort of 795 eligible participants. To explore alterations in the plasma metabolites that may be associated with CHD, two independent case-control groups were constructed based on strict coronary angiographic evidence: one group consisted of 150 CHD subjects (at least 1 major coronary artery with ≥ 80% stenosis and at least one stent implanted), and one group consisted of 150 age- and gender-matched control subjects (coronary arteries with ≤ 20% stenosis). The nontargeted plasma metabolomics profiling and coronary angiography data from these 300 subjects were analysed. Information regarding the traditional risk factors (TRFs) of CHD was collected from a clinical database and analysed by the study physicians. The Ethics Committee of Fuwai Hospital approved this study, which complies with the Declaration of Helsinki. Before collection of blood samples, all study participants provided written informed consent. The study strategy is shown in Fig. 1.

### Serum collection and preparation

Fasting plasma samples were collected in heparinized tubes before cardiac catheterization, chilled to 4 °C, centrifuged within 1 hour of collection (10 min at 2,300 rpm), separated into aliquots, and stored at −80 °C. The serum samples were then thawed at 4 °C, and 100 μL of serum from each sample was extracted with 400 μL of methanol using the Bravo liquid handling system (Agilent Technologies, USA). After vortexing for 2 min and incubating for 1 hour at −20 °C to precipitate the proteins, the mixture was then centrifuged at 12,000 rpm for 15 min at 4 °C. The supernatants were transferred to test vials and stored at −80 °C until the UHPLC-QTOF/MS analysis.

By mixing equal amounts of all 300 plasma samples, quality control (QC) samples were created to monitor the stability between the analytical batches and ensure data normalization. These QC samples were processed in the same manner as the testing samples.

### UHPLC-QTOF/MS analysis

The plasma samples were randomly injected for UHPLC-QTOF/MS analysis in both the electrospray positive and negative modes. Blank samples (75% ACN in water) and QC samples were injected between every eight samples during the acquisition. A UHPLC system (1290 series, Agilent Technologies, USA) coupled to a quadruple time-of-flight (QTOF) mass spectrometer (Agilent 6550 iFunnel QTOF, Agilent Technologies, USA) was employed for the primary metabolic detection and determination. Tandem mass spectrometry (MS/MS) was performed using another QTOF mass spectrometer (Triple TOF 5600+, AB SCIEX, USA), which was better at performing a qualitative metabolite analysis. The QC samples were used for the MS/MS data acquisition. Four mass range segments, 50–300 Da, 290–600 Da, 590–900 Da, and 890–1200 Da, were used to expand the coverage of the MS/MS spectra. The acquired MS/MS spectra were matched with the in-house MS/MS spectral library (Shanghai Institute of Organic Chemistry), which has been verified by the metabolite standards for metabolite identification (details are described in the Supplementary Information).

### Data pre-processing and annotation

ProteoWizard was employed to transform the MS raw data (.d) files to the mzXML format. The R package XCMS (version 3.2) was used to sequentially process the data. A data matrix that consisted of the retention time (RT), mass-to-charge ratio (m/z) values, and peak intensity was generated. The R package CAMERA was employed for peak annotation after XCMS data processing^13^. To obtain consistent variables, the metabolic matrix was further reduced by removing peaks with more than 80% missing values. A LOESS regression model based on the intensity of the metabolites in the QC samples was built to normalize the metabolite peaks in the test samples^14^.

### Statistical analysis

Continuous variables were compared using Student’s t-test, while categorical variables were compared using analysis of variance (ANOVA). For the comparison of CHD and control subjects, we ran multiple linear regression analyses that were adjusted for the TRFs of CHD (age, sex, body mass index (BMI), hypertension, diabetes, hyperlipidaemia, family history of CHD and smoking) and for the metabolite batch. The beta (β) value and 95% confidence interval (95% CI) were estimated. We also used a conservative Bonferroni correction to mitigate type I error in multiple testing, resulting in a significant threshold of 8.2 × 10^−5^ (0.05/611). To further quantify the independent correlations between significant metabolites and CHD, a logistic regression model was constructed. All of the analyses were performed using SAS version 9.4. P values < 0.05 were considered to be statistically significant.

### Data availability

The datasets generated and/or analysed in the current study are available from the corresponding author upon reasonable request.

## Results

### Demographic of the study population

The baseline characteristics and TRFs of the 150 CHD cases and 150 age- and gender-matched controls are displayed in Table 1. The average age of CHD patients was 55.24 ± 10.16 years and that of control subjects was 55.23 ± 10.23 years. Additionally, 50% of patients were female. As expected, CHD patients had significantly higher incidences of hypertension, diabetes, and hyperlipidaemia and more likely had a family history of CHD and smoker status.

**Table 1:** Baseline characteristics of the study population.

	Case group	Control group	P value
No. of subjects	150	150	
Females, %	50	50	0.9709
Age (years)	55.24 ± 10.16	55.23 ± 10.23	0.9910
BMI (kg/m^2^)	26.25 ± 4.01	26.24 ± 4.18	1.0000
Diabetes, %	40.7	12.7	0.0000
Hypertension, %	62.0	51.3	0.0001
Hyperlipidaemia, %	81.3	66.0	0.0024
Smokers, %	46.0	33.3	0.0247
Family history of CAD, %	32.7	20.0	0.0124
Extent of CHD			0.0000
No significant CAD, %	0	100	
Single vessel disease, %	14.7	0	
Double vessel disease, %	29.3	0	
Triple vessel disease, %	56	0	

The values are presented as mean ± SD or as percentages.

### Metabolic perturbation profile of CHD

**Figure 2:**
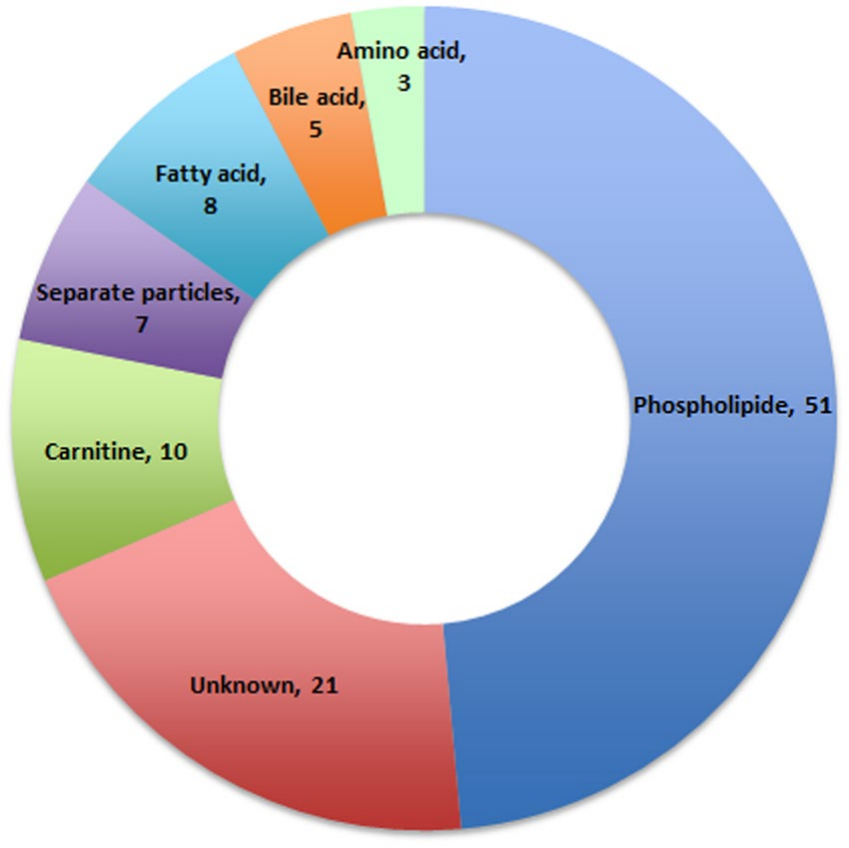
Metabolic perturbation profile of CHD. A total of 105 metabolites were significantly altered in CHD cases compared to angiographically normal controls. Each metabolite super-pathway is represented in a different colour.

The levels of 611 fasting plasma metabolites (466 in the positive mode (ESI+) and 145 in the negative mode (ESI−)) were obtained. After adjusting for the TRFs of CHD (age, sex, BMI, hypertension, diabetes, hyperlipidaemia, family history of CHD, and smoking) and for the metabolite batch using multiple linear regression models, 105 of the 611 metabolites were significantly different between the CHD cases and controls. As depicted in Fig. 2, the 105 metabolites (84 of which are known and 21 of which are unknown) could be classified into several principal classes: 51 are phospholipids, 10 are carnitines, 8 are free fatty acids, 5 are bile acid metabolites, 3 are amino acids and 7 are separate particles. The β value and 95% CI were collected, and these results are shown in Supplementary Table S1.

### Metabolites exhibiting the strongest association with CHD

To avoid type I error in multiple testing, a total of 6 metabolites out of the 105 differential metabolites mentioned above were identified with a Bonferroni-corrected cut-off of 8.2 × 10^−5^ (0.05/611), including palmitic acid (β = 0.205; p < 0.0001), linoleic acid (β = 0.133; p < 0.0001), 4-pyridoxic acid (β = 0.142; p < 0.0001), phosphatidylglycerol (20:3/2:0) (β = 0.287; p < 0.0001), carnitine (14:1) (β = 0.332; p < 0.0001), and lithocholic acid (β = 0.224; p < 0.0001). Their positive independent correlations with CHD were further quantified using a logistic regression model: palmitic acid (odds ratio [OR]: 7.24; 95% CI: (2.39, 21.912); P = 0.0001), linoleic acid (OR: 6.13; 95% CI: (2.367, 15.893); P = 0.0005), 4-pyridoxic acid (OR: 4.879; 95% CI: (2.158, 11.035); P = 0.0002), phosphatidylglycerol (20:3/2:0) (OR: 3.938; 95% CI: (1.891, 8.204); P = 0.0001), carnitine (14:1) (OR: 3.379; 95% CI: (1.913, 5.967); P < 0.0001), and lithocholic acid (OR: 2.791; 95% CI: (1.74, 4.477); P = 0.0003) (Table 2).

**Table 2:** List of metabolites with the strongest associations with CHD.

Metabolite	Super-pathway	Sub-pathway	Multiple linear regression analysis					Logistic regression analysis	
			Controls	CHD cases			↑/↓	OR (95% CI)^a^	P value^c^
				β^a^	95% CI^a^	P value^b^			
Palmitic acid	Fatty acids and conjugates	Saturated fatty acids	Ref.	0.205	(0.105,0.304)	3.64E-05	↑	7.237(2.39,21.912)	1 × 10^−4^
Linoleic acid	Fatty acids and conjugates	Unsaturated fatty acids	Ref.	0.133	(0.065,0.2)	7.67E-05	↑	6.133(2.367,15.893)	5 × 10^−4^
4-Pyridoxic acid	Pyridines and derivatives	Pyridinecarboxylic acids	Ref.	0.142	(0.071,0.212)	9.24E-05	↑	4.879(2.158,11.035)	2 × 10^−4^
Phosphatidylglycerol (20:3/2:0)	Glycerophospholipids	Phosphatidylglycerols	Ref.	0.287	(0.145,0.43)	7.65E-05	↑	3.938(1.891,8.204)	1 × 10^−4^
Carnitine (14:1)	Quaternary ammonium salts	Carnitines	Ref.	0.332	(0.187,0.477)	6.39E-06	↑	3.379(1.913,5.967)	<1 × 10^−4^
Lithocholic acid	Steroids and steroid derivatives	Bile acids, alcohols and derivatives	Ref.	0.224	(0.112,0.336)	6.78E-05	↑	2.791(1.74,4.477)	3 × 10^−4^

^a^The models are adjusted for age, sex, body mass index (BMI), hypertension, diabetes, hyperlipidaemia, family history of CHD, smoking and metabolite batch. ^b^Conservative Bonferroni correction to a significant threshold of 8.2 × 10^−5^ (0.05/611) was performed. ^c^A P value < 0.05 was considered statistically significant. The arrows ↑/↓, respectively, indicate an increase or a decrease of the metabolite levels in the plasma of CHD patients compared to those of control subjects.

## Discussion

Using a nontargeted metabolomics approach, we explored the molecular perturbation profile of patients with severe CHD compared with angiographically normal controls. We identified 105 metabolites that were distinctly altered in plasma samples, including 51 phospholipids, 10 carnitines, 8 free fatty acids, 5 bile acid metabolites, 3 amino acids and 7 separate particles. Of these, six metabolites were discovered as having the strongest correlations with CHD after adjusting for multiple testing (the adjusted ORs ranged from 2.79 to 7.24); to the best of our knowledge, the correlations of CHD with 4-pyridoxic acid, lithocholic acid and phosphatidylglycerol (20:3/2:0) were first reported in this study. The links between metabolic dysregulation and CHD that were detected in our analysis not only verified the well-established implications about lipids but also identified novel metabolic targets that are potentially involved in CHD pathogenesis. Here, we theoretically estimated the assumed causality between significant metabolic perturbations and CHD and provided insight on the internal mechanisms that may be involved.

### Palmitic acid

Of the six identified metabolites, palmitic acid had the strongest positive correlation with CHD (OR: 7.24; 95% CI: (2.39, 21.912); P = 0.0001), which was consistent with previous findings^15,16^. As the most abundant saturated fatty acid (SFA), palmitic acid (16:0) accounts for nearly 50% of the total daily SFA intake. Although the association between SFA intake and CHD risk is debated^17^, strong positive associations have been identified between CHD development and palmitic acid intake^18,19^. A recently updated meta-analysis of clinical trials reported that palmitic acid (16:0) significantly increased total cholesterol levels and low-density lipoprotein (LDL) cholesterol levels^20^. Furthermore, an *in vitro* experiment confirmed that palmitic acid could induce vascular endothelial dysfunction by triggering inflammatory signals and provided novel insight on the mechanisms underlying the correlation between palmitic acid and atherosclerosis^21^.

### Linoleic acid

Linoleic acid (LA) levels were elevated in severe CHD patients compared with control subjects in our study. Each integer increase in the LA level is associated with a 6.13-fold increase (95% CI: (2.367, 15.893); P = 0.0005) in CHD risk. As the most abundant polyunsaturated fatty acid (PUFA), omega 6 (n-6) LA is usually confused with general PUFAs or with omega 3 (n-3) PUFAs when explaining clinical findings of its relationship with the risk of cardiovascular disease. Meanwhile, a comprehensive randomized controlled trial discovered that the well-known atheroprotective properties of PUFA intake are specifically attributable to n-3 PUFAs and are not relevant to n-6 LA^22^. Moreover, they found that increased LA intake was associated with higher risks of all-cause death, CHD, and cardiovascular disease^23^. This may explain the positive connection between the plasma LA level and CHD found in our study. The mechanism linking an increased LA level to cardiovascular pathogenesis has also been confirmed. Researchers found that an increased level of linoleic acid resulted in increased oxidized LA metabolites (OXLAMs), which can induce the formation of macrophage foam cells and endothelial cell dysfunction^24,25^. Another study suggests that LA may also activate the NF-κB pathway and TNF-mediated endothelial cell dysfunction, which play critical roles in the pathogenesis of atherosclerosis^26^.

### Pyridoxic acid

The third metabolite found to be associated with CHD in this study is pyridoxic acid, which is the end product of vitamin B6 catabolism. Three forms of Vitamin B6 are typically detected in the plasma: pyridoxal 5′-phosphate (PLP), pyridoxal (PL), and 4-pyridoxic acid (4-PA). PLP is the biochemically active form of Vitamin B6 and is an essential coenzyme in almost all living systems^27^. Pyridoxic acid has received little attention in cardiovascular research. Meanwhile, a number of previous studies have confirmed that lower circulating PLP concentrations or lowering vitamin B6 intake is associated with an increased risk of CHD, hypertension, diabetes or stroke^28–31^. What is often overlooked is that low circulating PLP levels have been observed in combination with an increase in plasma 4-PA levels under inflammatory conditions^32–34^. To our knowledge, our study was the first to confirm a positive association between the plasma 4-PA level and CHD risk (OR: 4.879; 95% CI: (2.158, 11.035); P = 0.0002). Several hypotheses have been suggested to explain the inverse changes shown in B6 vitamins during inflammation-related pathogenesis. Some studies have suggested the mobilization of PLP from the plasma to the site of inflammation (erythrocytes or affected tissues) for use of PLP-dependent enzymes^34,35^. Other studies have indicated a higher catabolic rate of PLP under oxidative stress and activated cellular immunity conditions^33,36^. A study also proposed the PA:(PL + PLP) ratio (PAr) as an indicator of vitamin B6 catabolism and confirmed that the PAr is a predictor for all-cause mortality^37^. A possible inhibitory effect of 4-PA on vitamin B6 metabolism has also been discussed. Experimental data have shown the inhibitory effect of 4-PA on PL kinase^37,38^. Additionally, an observational study showed that the plasma 4-PA level was a stronger covariate with homocysteine (an independent CHD risk factor) than the plasma PLP level^39^. In conclusion, it is likely that CHD pathogenesis may cause an increase in the 4-PA level, but a perturbed 4-PA status may contribute to the development of more severe inflammation as well.

### Phosphatidylglycerol (20:3/2:0)

A strong correlation of phosphatidylglycerol (20:3/2:0) with CHD was identified in our study. Since hundreds of different glycerophospholipids have been observed in plasma lipoproteins (Lp (a)) and atherosclerotic plaques, our finding was not unexpected^40^. However, recent studies have suggested that the function of glycerophospholipids might different depending on the cell type that is activated, the oxidation/inflammation status, the subclass of glycerophospholipid or the species of the bound fatty acids^16,40^. Some glycerophospholipids may act as antioxidants^41,42^, whereas others may induce pro-atherogenic effects on endothelial cells^43,44^. Regarding phosphatidylglycerol (PGs), which a phosphoglycerol moiety occupies a glycerol substitution site, little evidence has been shown on their relationship with CHD, and we were the first to reveal a positive relationship between PG**(**20:3/2:0) and CHD (OR: 3.938; 95% CI: (1.891, 8.204); P = 0.0001) in human plasma. Several previous studies have provided evidence that PGs are significantly increased in atherosclerotic rats^45,46^. Elevated levels of PGs were also found in patients with type 2 diabetes and prediabetes^47^. PGs are precursors for another glycerophospholipid, cardiolipin (CL). Thus, the association of oxidant stress and mitochondrial dysfunction with CL may suggest a pro-atherogenic role for PGs^48^. Meanwhile, the involvement of PGs in endogenous antioxidant defence mechanisms has also been proposed, as PGs are the second-most abundant phospholipid in lung surfactant and play an anti-inflammatory role during infection^49^.

### Acylcarnitine (14:1)

Almost all metabolomics studies concerning CHD thus far have mentioned acylcarnitines^6,9,10^. We identified acylcarnitine (14:1), a medium-chain dicarboxylated acylcarnitine, as being an important metabolite associated with CHD (OR: 3.379; 95% CI: (1.913, 5.967); P < 0.0001). As expected, the medium-chain acylcarnitine cluster has been reported as being associated with cardiovascular diseases in multiple studies of different designs^50^. The well-known metabolic function of acylcarnitines is to transport long-chain fatty acids into the mitochondria for β-oxidation, which is how the heart acquires most of its energy. Therefore, the accumulation of acylcarnitines, especially those of medium-chain species, has been attributed to incomplete fatty acid oxidation and energy metabolism in CHD^51^. Studies have also suggested the elevation of acylcarnitines as being a signature for inadequate β-oxidation in macrophage lipid metabolism^52^, which is a key process underlying atherosclerosis. Moreover, L-carnitine and acylcarnitines have been shown to contribute to the development of atherosclerosis by increasing the levels of the independent CHD risk factor trimethylamine N-oxide (TMAO) in experimental animals^53^. Another study also showed that decreasing the levels of L-carnitine and its derivatives can attenuate atherosclerosis pathogenesis^54^.

### Lithocholic acid

Lithocholic acid (LCA) belongs to the bile acid family, which is composed of molecules that are essential for the absorption of dietary fats and the regulation of cholesterol homeostasis. Our study was the first to report a relationship between elevated plasma lithocholic acid levels and CHD (OR: 2.791; 95% CI: (1.74, 4.477); P = 0.0003). Faecal metabolomics studies have shown that CHD patients are unable to excrete adequate amounts of bile acids to remove excess serum cholesterol^55^. This can lead to an accumulation of plasma LCA, which we detected in CHD patients in our study. Researchers who observed that elevated lithocholic acid levels could induce hepatic inflammation in cholestasis also uncovered the vascular inflammatory role of circulating lithocholic acid. Experimental evidence showed that elevated LCA levels might cause endothelial dysfunction via the production of reactive oxygen species (ROS) by the induction of the NF-κB and p38 MAPK signalling pathways, which suggested a potential pro-atherogenic role for LCA^56^. However, several studies have provided conflicting data on the relationship between LCA and coronary atheroma^57^, which reminds us that this field of study is worthy of further investigation.

### Strengths of the current study

We employed a nontargeted metabolomics approach that not only confirmed previous findings from targeted assays but also revealed a much wider metabolite spectrum that is related to CHD. We mainly explored the CHD metabolomics profile in Chinese populations. There are very large differences in both the prevalence and levels of risk factors for coronary heart disease among different ethnic groups^58,59^, and previous CHD metabolomics studies were predominately conducted on Caucasian populations^6–11^. Thus, it is important to develop ethnicity-specific CHD metabolomics analyses to more accurately determine the underlying metabolic perturbations involved in CHD pathogenesis. In addition, we optimized the high-resolution mass spectrometry (HRMS) process by combining two QTOF mass spectrometers, the Agilent 6550 iFunnel QTOF and Triple TOF 5600+. The Agilent 6550 iFunnel QTOF is better for metabolite detection and determination, while the Triple TOF 5600+ is better for qualitative metabolite analyses. Furthermore, the study cohort was screened with very restrictive exclusion criteria and strictly grouped based on angiographic evidence to reduce variations in the levels of the plasma metabolites.

### Limitations of the current study

First, the current study is a case-control study that predominately reveals the association between metabolite signatures and CHD status. However, the causal relationship and the internal mechanisms of metabolite signatures and CHD status are still unclear. Therefore, a prospective nontargeted study is recommended in the future. Second, our results lack the verification of a targeted analysis. An independent cohort is required to further replicate and confirm our findings. Moreover, as the metabolic fingerprinting assessment was conducted at a single time point, this study is unable to trace back to the disturbed metabolic flux in CHD patients. Additional isotope tracer techniques, especially those used in animal models, are recommended to complement the techniques used in the current study.

## Conclusions

Overall, our study examined the global plasma metabolomics profile related to CHD by employing a nontargeted UHPLC-QTOF/MS metabolomics approach. We not only confirmed common alterations in plasma metabolism but also identified 3 novel metabolites (4-pyridoxic acid, PG (20:3/2:0) and lithocholic acid) that exhibited strong correlations with CHD. These findings provide further insight into the pathogenic mechanisms of CHD and may lead to improved diagnostic and therapeutic options for patients with CHD. Further targeted studies based on the metabolic perturbations that we reported are warranted.

## Electronic supplementary material

Supplementary Information
