# Supplementary material for: Investigation of novel metabolites potentially involved in the pathogenesis of coronary heart disease using a UHPLC-QTOF/MS-based metabolomics approach
Source: Sci Rep. 2017 Nov 10;7:15357. doi: 10.1038/s41598-017-15737-3 (PMC5681629; doi:10.1038/s41598-017-15737-3)
Supplement: Supplementary file 1 — Supplementary Information [file 41598_2017_15737_MOESM1_ESM.pdf]

Supplementary Information

Investigation of novel metabolites potentially involved in the pathogenesis of coronary heart disease using a UHPLC-QTOF/MS-based metabolomics approach

Yiping Li[1], Dong Zhang[1], Yuan He[1], Changzhe Chen[1], Chenxi Song[1], Yanyan Zhao[1], Yinxiao Bai[1], Yang Wang[1], Jielin Pu[1], Jingzhou Chen[1], YuejinYang[1], Kefei Dou[1]*

[1]State Key Laboratory of Cardiovascular Disease, Fuwai Hospital, National Centre for Cardiovascular Diseases, Chinese Academy of Medical Sciences and Peking Union Medical College, Beijing, 100037, People's Republic of China

*Corresponding author. Tel: +86 10 88396594; Email: drdoukefei@126.com

Key Words: coronary heart disease, nontargeted metabolomics, UHPLC-QTOF/MS, plasma, 4-pyridoxic acid, lithocholic acid, phosphatidylglycerol (20:3/2:0)

UHPLC-QTOF/MS analysis method details

Waters ACQUITY UHPLC HSS T3 columns [particle size, 1.8μm; 100 mm (length) × 2.1 mm (i.d.)] were employed for the LC separation. Parameters in details were set as follows: column temperature: 25 °C; sample injection volume: 6μL; flow rate: 0.5 mL/min. The mobile phases A was 0.1 % FA in water in positive mode (ESI+) or 0.5 mM NH_4F in water in negative mode (ESI-). The mobile phase B was 0.1 % FA in ACN in positive mode or 100 % ACN in negative mode. The linear gradient was as follows: 0–1min: 1% B, 1–8min: 1%B to 100% B, 8–10min: 100% B, 10–10.1min: 100% B to 1% B, 10.1–12min: 1 % B. The TOF mass range was among m/z 50–1200 Da and the acquisition rate was 4 spectra/s. The parameters of MS data acquisition were as follows: sheath gas temperature: 400 ℃; dry gas temperature: 250 ℃; dry gas flow: 16 L/min; sheath gas flow: 12 L/min; capillary voltage: 3000 V in positive mode or -3000 V in negative mode; nebulizer pressure: 20 psi in positive or 40 psi in negative mode and nozzle voltage: 0 V.

Metabolite identification

AB Sciex TripleTOF 5600+, another quadruple time-of-flight mass spectrometer, was used to seek tandem mass spectrometry (MS/MS) spectra data for metabolites identification. QC samples were employed for MS/MS data acquisition. The acquired MS/MS spectra were matched with in-house standard MS/MS spectral library of Shanghai Institute of Organic Chemistry verified by metabolite standards. Parameters in details were set as follows: GAS1, 60; GAS2: 60; TEM: 600 ℃; CUR: 30; ISVF: 5500 V in positive mode or -4500 V in negative mode respectively. The MS/MS spectra match score was calculated using dot-product algorithm ranging from 0-1. The cutoff for match score was set as 0.8. Manually checked method was also employed to confirm the identification in MS/MS spectra matching. All m/z errors are controlled within 25 ppm and all the RT errors are less than 18 seconds.

Supplementary table S1：Identification of 105 metabolites significantly altered in CHD patients

Ionization mode	Adduct	RT (s)	m/z	Metabolite	Super-pathway	Sub-pathway	Multiple linear regression analysis				↑/↓
							Control	CHD case			
								β[a]	95%CI[a]	P-value[a]	
ESI (-)	[M+2H-CH4] 2+ 246.12	169.372	116.0499	Indole	Organoheterocyclic compounds	Indoles and derivatives	Ref.	-0.082	(-0.163,0)	0.0489	↓
ESI (+)	NA	182.232	118.0652	Unknown	Unknown	Unknown	Ref.	-0.063	(-0.115,-0.011)	0.0225	↓
ESI (-)	[M+H]+ 128.044	145.443	129.0551	alpha-Ketocaproic acid	Keto acids and derivatives	Medium-chain keto acids and derivatives	Ref.	-0.12	(-0.232,-0.009)	0.0421	↓
ESI (-)	[M+H]+ 153.057	42.000	154.0619	L-Histidine	Amino acids, peptides, and analogues	Histidine and derivatives	Ref.	-0.122	(-0.244,0)	0.0416	↓
ESI (+)	[M+K+NH_3]+ 126.092<br>[M+Na+ NH_3]+ 142.066<br>[M+H+ NH_3]+164.048	109.486	182.0816	L-Tyrosine	Amino acids, peptides, and analogues	Tyrosine and derivatives	Ref.	-0.09	(-0.159,-0.022)	0.0129	↓
ESI (+)	NA	62.775	184.0578	4-Pyridoxic acid	Pyridines and derivatives	Pyridinecarboxylic acids	Ref.	0.205	(0.105,0.304)	0.0000	↑
ESI (-)	[3M+2Na+K-H]2+ 102.05	147.083	195.0524	Salicyluric acid	Benzene and substituted derivatives	Hippuric acids	Ref.	-0.775	(-1.404,-0.146)	0.0150	↓
ESI (+)	NA	198.607	195.0884	Caffeine	Organoheterocyclic compounds	Purines and purine derivatives	Ref.	-0.615	(-1.205,-0.024)	0.0499	↓
ESI (+)	[M+H]+ 203.116	59.986	204.1238	Acetylcarnitine	Quaternary ammonium salts	Carnitines	Ref.	0.06	(0.004,0.117)	0.0290	↑
ESI (+)	[M+Na+ NH_3]+ 168.123	243.012	208.1310	Caprylic acid	Fatty acids and conjugates	Saturated fatty acids	Ref.	0.355	(0.121,0.588)	0.0029	↑
ESI (+)	NA	362.315	226.1226	Hydroxybutyrate	Fatty Acids and Conjugates	Hydroxy fatty acids	Ref.	0.384	(0.105,0.664)	0.0089	↑
ESI (+)	NA	236.771	248.0209	DL-O-tyrosine	Amino acids, peptides, and analogues	Tyrosine and derivatives	Ref.	-0.824	(-1.482,-0.166)	0.0183	↓
ESI (-)	NA	538.183	255.2331	Unknown	Unknown	Unknown	Ref.	0.083	(0.024,0.141)	0.0050	↑
ESI (-)	NA	646.742	255.2327	Palmitic acid	Fatty acids and conjugates	Saturated fatty acids	Ref.	0.133	(0.065,0.2)	0.0001	↑

ESI (+)	[M+K]+ 221.222 [M+Na]+ 237.196	224.412	260.1857	carnitine(6:0)	Quaternary ammonium salts	Carnitines	Ref.	0.217	(0.069,0.365)	0.0041	↑
ESI (-)	[M+H]+ 276.21	464.492	277.2159	alpha-Linolenic acid	Fatty acids and conjugates	Unsaturated fatty acids	Ref.	0.469	(0.027,0.911)	0.0387	↑
ESI (-)	[M+K]+ 238.256 [M+Na]+ 254.224	484.479	277.2169	cis-(6,9,12)-Linolenic acid	Fatty acids and conjugates	Unsaturated fatty acids	Ref.	0.27	(0.126,0.413)	0.0003	↑
ESI (-)	[M+Na]+ 256.248 [M+H]+ 278.229	511.259	279.2327	Linoleic acid	Fatty acids and conjugates	Unsaturated fatty acids	Ref.	0.142	(0.071,0.212)	0.0001	↑
ESI (-)	NA	646.987	279.2324	Unknown	Unknown	Unknown	Ref.	0.217	(0.12,0.315)	0.0000	↑
ESI (-)	[M+K]+ 242.28 [M+H]+ 280.235	514.423	281.2400	trans-Vaccenic acid	Fatty acids and conjugates	Unsaturated fatty acids	Ref.	0.125	(0.042,0.208)	0.0027	↑
ESI (-)	NA	575.276	281.2483	Unknown	Unknown	Unknown	Ref.	0.128	(0.033,0.223)	0.0098	↑
ESI (-)	NA	646.498	281.2483	Oleic acid	Fatty acids and conjugates	Unsaturated fatty acids	Ref.	0.24	(0.118,0.362)	0.0001	↑
ESI (+)	[M+H]+ 287.211	268.688	288.2168	carnitine(8:0)	Quaternary ammonium salts	Carnitines	Ref.	0.23	(0.035,0.425)	0.0259	↑
ESI (+)	NA	268.688	289.2202	Testosterone	Steroids and steroid derivatives	Androgens and derivatives	Ref.	0.231	(0.018,0.444)	0.0416	↑
ESI (+)	NA	233.682	333.1328	4-Methoxyphenylacetic acid	Benzenoids	Anisoles	Ref.	0.239	(0.014,0.465)	0.0326	↑
ESI (+)	NA	308.500	342.2142	Unknown	Unknown	Unknown	Ref.	0.115	(0.003,0.227)	0.0398	↑
ESI (+)	[M+H-CH2]+ 357.28	335.771	344.2792	carnitine(12:0)	Quaternary ammonium salts	Carnitines	Ref.	0.294	(0.101,0.488)	0.0032	↑
ESI (+)	NA	333.890	370.2854	Unknown	Unknown	Unknown	Ref.	0.287	(0.145,0.43)	0.0001	↑
ESI (+)	[M+Na+HCOOH]+ 301.297	350.614	370.2947	carnitine(14:1)	Quaternary ammonium salts	Carnitines	Ref.	0.332	(0.187,0.477)	0.0000	↑
ESI (-)	NA	523.509	375.2908	Lithocholic acid	Steroids and steroid derivatives	Bile acids, alcohols and derivatives	Ref.	0.46	(0.274,0.646)	0.0000	↑
ESI (-)	[M+H-CH2]+ 404.293	470.408	391.2850	Deoxycholic acid	Steroids and steroid derivatives	Bile acids, alcohols and	Ref.	0.159	(0.04,0.278)	0.0073	↑

						derivatives					
ESI (+)	NA	376.335	398.3261	carnitine(16:1)	Quaternary ammonium salts	Carnitines	Ref.	0.143	(0.031,0.255)	0.0100	↑
ESI (+)	[M+H-C2H4]+ 427.371	396.648	400.3417	L-Palmitoylcarnitine	Quaternary ammonium salts	Carnitines	Ref.	0.093	(0.02,0.167)	0.0082	↑
ESI (-)	NA	397.082	407.2791	Cholic acid	Steroids and steroid derivatives	Bile acids, alcohols and derivatives	Ref.	0.19	(0.039,0.342)	0.0075	↑
ESI (-)	NA	251.974	413.1996	PG(11:0/0:0)	Glycerophospholipids	Phosphatidylglycerols	Ref.	0.209	(0.071,0.348)	0.0023	↑
ESI (+)	[M+H]+ 421.322	369.319	422.3259	Unknown	Unknown	Unknown	Ref.	0.102	(0.008,0.196)	0.0415	↑
ESI (+)	[M+H]+ 423.333	385.197	424.3416	carnitine(18:2)	Quaternary ammonium salts	Carnitines	Ref.	0.084	(0,0.167)	0.0488	↑
ESI (+)	NA	384.715	426.3478	carnitine(18:1)	Quaternary ammonium salts	Carnitines	Ref.	0.087	(0.001,0.173)	0.0483	↑
ESI (+)	NA	403.342	426.3575	Unknown	Unknown	Unknown	Ref.	0.159	(0.043,0.275)	0.0047	↑
ESI (-)	NA	349.082	432.3184	Glycolithocholic acid	Steroids and steroid derivatives	Bile acids, alcohols and derivatives	Ref.	0.186	(0.057,0.315)	0.0040	↑
ESI (+)	[M+2Na-H]+ 405.398 [M+Na]+ 427.371	395.082	450.3570	carnitine(20:3)	Quaternary ammonium salts	Carnitines	Ref.	0.104	(0.002,0.206)	0.0396	↑
ESI (-)	NA	411.217	452.2774	PE(16:0/0:0)	Glycerophospholipids	phosphoethanolamines	Ref.	-0.16	(-0.293,-0.028)	0.0152	↓
ESI (+)	NA	382.211	460.2688	PC(O-6:0/0:0)	Glycerophospholipids	Phosphatidylcholines	Ref.	-0.095	(-0.16,-0.03)	0.0034	↓
ESI (+)	[M+H-COCH2]+ 501.285	399.818	460.2811	Unknown	Unknown	Unknown	Ref.	-0.104	(-0.2,-0.009)	0.0172	↓
ESI (+)	NA	442.224	468.3080	PC(14:00:0)	Glycerophospholipids	Phosphatidylcholines	Ref.	-0.281	(-0.477,-0.086)	0.0026	↓
ESI (+)	[M+Na]+ 453.286	416.539	476.2742	PE(16:00:0)	Glycerophospholipids	phosphoethanolamines	Ref.	-0.137	(-0.207,-0.066)	0.0001	↓
ESI (-)	NA	395.863	478.2839	PE(P-16:0/2:0)	Glycerophospholipids	phosphoethanolamines	Ref.	-0.129	(-0.257,-0.002)	0.0323	↓
ESI (+)	NA	399.800	478.2926	PE(18:20:0)	Glycerophospholipids	phosphoethanolamines	Ref.	-0.103	(-0.185,-0.021)	0.0066	↓
ESI (+)	NA	399.707	480.2982	PE(18:10:0)	Glycerophospholipids	phosphoethanolamines	Ref.	-0.106	(-0.184,-0.028)	0.0038	↓
ESI (+)	NA	469.839	482.3237	PC(15:00:0)	Glycerophospholipids	Phosphatidylcholines	Ref.	-0.11	(-0.216,-0.003)	0.0291	↓
ESI (+)	NA	460.382	494.3592	PC(O-17:10:0)	Glycerophospholipids	Phosphatidylcholines	Ref.	-0.253	(-0.437,-0.07)	0.0048	↓

ESI (-)	NA	396.600	502.2835	PE(20:3/0:0)	Glycerophospholipids	phosphoethanolamines	Ref.	0.17	(0.046,0.294)	0.0095	↑
ESI (+)	[M+H]+ 501.285	400.719	502.2930	Unknown	Unknown	Unknown	Ref.	0.117	(0.046,0.188)	0.0017	↑
ESI (+)	[M+H-H20]+ 519.331	404.724	502.3271	PC(O-16:10:0)	Glycerophospholipids	Phosphatidylcholines	Ref.	-0.108	(-0.186,-0.03)	0.0038	↓
ESI (+)	[M+Na]+ 479.298	429.402	502.2896	Unknown	Unknown	Unknown	Ref.	-0.103	(-0.182,-0.024)	0.0041	↓
ESI (+)	[M+H+ NH_3]+ 500.29<br>[M+H]+ 517.316	381.454	518.3237	PC(18:30:0)	Glycerophospholipids	Phosphatidylcholines	Ref.	-0.143	(-0.25,-0.037)	0.0040	↓
ESI (+)	[M+H]+ 519.331	404.067	520.3399	PC(18:20:0)	Glycerophospholipids	Phosphatidylcholines	Ref.	-0.085	(-0.135,-0.035)	0.0005	↓
ESI (+)	NA	549.386	520.3438	Unknown	Unknown	Unknown	Ref.	0.23	(0,0.461)	0.0469	↑
ESI (+)	NA	404.071	522.3460	PC(18:10:0)	Glycerophospholipids	Phosphatidylcholines	Ref.	-0.066	(-0.119,-0.013)	0.0077	↓
ESI (+)	[M+Na]+ 501.285	400.574	524.2742	PE(20:40:0)	Glycerophospholipids	phosphoethanolamines	Ref.	0.113	(0.044,0.181)	0.0017	↑
ESI (-)	NA	218.255	525.2690	PA(4:0/20:5)	Glycerophospholipids	Diacylglycerophosphates	Ref.	0.496	(0.227,0.765)	0.0003	↑
ESI (-)	[3M+2H]2+ 352.192<br>[M+K+NaCOOH]+ 422.35<br>[M+K+HCOOH]+ 444.33	516.602	529.3107	PA(6:0/18:3)	Glycerophospholipids	Diacylglycerophosphates	Ref.	0.267	(0.104,0.43)	0.0008	↑
ESI (+)	[M+Na+ NH_3]+ 500.29<br>[M+Na]+ 517.316	381.296	540.3055	Unknown	Unknown	Unknown	Ref.	-0.129	(-0.225,-0.032)	0.0043	↓
ESI (+)	[M+H-COCH2]+ 581.305	404.327	540.3150	Unknown	Unknown	Unknown	Ref.	-0.339	(-0.567,-0.11)	0.0027	↓
ESI (+)	[M+Na]+ 537.341	332.556	560.3319	PC(12:0/6:0)	Glycerophospholipids	Phosphatidylcholines	Ref.	0.126	(0.011,0.24)	0.0338	↑
ESI (+)	NA	405.002	566.3216	PC(20:40:0)	Glycerophospholipids	Phosphatidylcholines	Ref.	0.09	(0.005,0.174)	0.0361	↑
ESI (+)	[M+K]+ 535.331	332.028	574.2926	PS(17:1/2:0)	Glycerophospholipids	Phosphatidylserines	Ref.	0.11	(0.03,0.19)	0.0085	↑
ESI (+)	NA	404.070	574.2586	PS(16:1/3:0)	Glycerophospholipids	Phosphatidylserines	Ref.	-0.119	(-0.203,-0.034)	0.0049	↓
ESI (+)	NA	330.211	583.2543	PG(3:018:4)	Glycerophospholipids	Phosphatidylglycerols	Ref.	0.385	(0.169,0.601)	0.0003	↑

ESI (+)	NA	346.353	584.3317	PC(2:0/20:5)	Glycerophospholipids	Phosphatidylcholines	Ref.	0.331	(0.055,0.607)	0.0183	↑
ESI (+)	NA	330.266	585.2619	Bilirubin	Steroids and steroid derivatives	Bile acids, alcohols and derivatives	Ref.	0.24	(0.103,0.378)	0.0005	↑
ESI (+)	[M+Na]+ 563.365<br>[M+H]+ 585.34	351.675	586.3465	PC(2:0/20:4)	Glycerophospholipids	Phosphatidylcholines	Ref.	0.201	(0.007,0.395)	0.0498	↑
ESI (+)	[M+Na]+ 567.32	316.322	590.3060	PE(22:6/2:0)	Glycerophospholipids	phosphoethanolamines	Ref.	0.247	(0.095,0.4)	0.0018	↑
ESI (+)	[M+K+CF3COOH]+417.44<br>[M+H+CF3COOH]+455.39	453.711	590.3809	PC(2:020:2)	Glycerophospholipids	Phosphatidylcholines	Ref.	-0.136	(-0.242,-0.031)	0.0093	↓
ESI (+)	[3M+H+K]2+ 386.208<br>[M+Na]+ 576.324	298.774	599.3138	PG(20:3/2:0)	Glycerophospholipids	Phosphatidylglycerols	Ref.	0.224	(0.112,0.336)	0.0001	↑
ESI (+)	[M+Na]+ 581.305	404.188	604.2903	PE(22:6/3:0)	Glycerophospholipids	phosphoethanolamines	Ref.	-0.143	(-0.228,-0.058)	0.0006	↓
ESI (+)	[M+K+HCOOH]+ 521.346	435.361	606.3060	PC(20:5/2:0)	Glycerophospholipids	Phosphatidylcholines	Ref.	-0.088	(-0.171,-0.006)	0.0271	↓
ESI (+)	NA	330.410	607.2428	Unknown	Unknown	Unknown	Ref.	0.332	(0.128,0.536)	0.0010	↑
ESI (+)	NA	294.454	614.3048	PS(20:2/2:0)	Glycerophospholipids	Phosphatidylserines	Ref.	0.234	(0.111,0.357)	0.0002	↑
ESI (+)	[M+2K-H]+ 537.384<br>[M+K]+ 575.343	311.627	614.3050	PS(18:2/4:0)	Glycerophospholipids	Phosphatidylserines	Ref.	0.328	(0.108,0.548)	0.0049	↑
ESI (+)	[M+H+ NH_3]+ 616.417	464.425	634.4512	PC(3:0/22:1)	Glycerophospholipids	Phosphatidylcholines	Ref.	-0.074	(-0.14,-0.009)	0.0172	↓
ESI (+)	[M+H]+ 702.57	475.347	703.5746	SM(d16:018:1)	Sphingolipids	Sphingomyelins	Ref.	0.2	(0.003,0.397)	0.0470	↑
ESI (+)	[3M+2Na+2K-H]3+ 684.579	333.979	725.5558	SM(d14:120:0)	Sphingolipids	Sphingomyelins	Ref.	0.324	(0.023,0.625)	0.0336	↑
ESI (-)	NA	491.110	738.5074	PE(18:2/18:2)	Glycerophospholipids	phosphoethanolamines	Ref.	0.118	(0.025,0.211)	0.0133	↑
ESI (+)	[M+K]+ 741.596	387.453	780.5516	PC(16:018:2)	Glycerophospholipids	Phosphatidylcholines	Ref.	0.142	(0.037,0.247)	0.0076	↑

ESI (+)	[M+K]+ 741.596	409.226	780.5517	Unknown	Unknown	Unknown	Ref.	0.087	(0.015,0.158)	0.0128	↑
ESI (+)	[M+Na+K-H]+ 719.61	430.357	780.5518	Unknown	Unknown	Unknown	Ref.	0.079	(0.022,0.136)	0.0049	↑
ESI (+)	[M+H]+ 779.545	518.518	780.5515	Unknown	Unknown	Unknown	Ref.	0.238	(0.111,0.365)	0.0002	↑
ESI (+)	[M+Na]+ 757.564	547.901	780.5513	Unknown	Unknown	Unknown	Ref.	0.081	(0.004,0.158)	0.0304	↑
ESI (+)	NA	568.030	780.5515	Unknown	Unknown	Unknown	Ref.	0.283	(0.123,0.442)	0.0005	↑
ESI (+)	NA	548.333	782.5658	PC(14:022:4)	Glycerophospholipids	Phosphatidylcholines	Ref.	-0.12	(-0.209,-0.031)	0.0119	↓
ESI (+)	NA	594.114	782.5668	PC(12:024:4)	Glycerophospholipids	Phosphatidylcholines	Ref.	-0.121	(-0.236,-0.006)	0.0466	↓
ESI (+)	NA	592.031	786.5991	PC(10:026:2)	Glycerophospholipids	Phosphatidylcholines	Ref.	-0.131	(-0.256,-0.005)	0.0416	↓
ESI (+)	NA	430.376	792.5838	PC(P-18:0/20:5)	Glycerophospholipids	Phosphatidylcholines	Ref.	0.129	(0.005,0.252)	0.0375	↑
ESI (+)	NA	518.514	804.5515	PC(16:020:4)	Glycerophospholipids	Phosphatidylcholines	Ref.	0.157	(0.028,0.287)	0.0149	↑
ESI (+)	NA	567.765	804.5510	Unknown	Unknown	Unknown	Ref.	0.118	(0.013,0.222)	0.0217	↑
ESI (+)	NA	293.066	806.5650	PC(16:122:5)	Glycerophospholipids	Phosphatidylcholines	Ref.	0.143	(0.015,0.272)	0.0366	↑
ESI (+)	[M+Na+NaCOOH]+719.612	474.797	810.5999	PC(16:022:4)	Glycerophospholipids	Phosphatidylcholines	Ref.	-0.196	(-0.335,-0.058)	0.0065	↓
ESI (+)	[M+Na]+ 787.608	590.973	810.5981	PC(14:024:4)	Glycerophospholipids	Phosphatidylcholines	Ref.	-0.13	(-0.248,-0.013)	0.0254	↓
ESI (+)	NA	518.344	830.5648	PC(22:516:0)	Glycerophospholipids	Phosphatidylcholines	Ref.	0.178	(0.061,0.296)	0.0028	↑
ESI (+)	NA	547.899	830.5648	PC(20:418:1)	Glycerophospholipids	Phosphatidylcholines	Ref.	0.071	(0.004,0.138)	0.0301	↑
ESI (+)	NA	518.514	832.5819	PC(18:020:4)	Glycerophospholipids	Phosphatidylcholines	Ref.	0.149	(0.007,0.29)	0.0371	↑
ESI (+)	[M+H]+ 833.59	517.840	834.5956	PC(18:422:2)	Glycerophospholipids	Phosphatidylcholines	Ref.	0.15	(0.017,0.284)	0.0210	↑
ESI (+)	[M+Na]+ 833.59	518.365	856.5804	PC(20:5/22:4)	Glycerophospholipids	Phosphatidylcholines	Ref.	0.107	(0.001,0.213)	0.0475	↑
ESI (+)	[M+Na+NaCOOH]+781.565	547.897	872.5398	PS(24:4/17:2)	Glycerophospholipids	Phosphatidylserines	Ref.	0.103	(0.004,0.202)	0.0391	↑

a. The models are adjusted for age, sex, body mass index (BMI), hypertension, diabetes, hyperlipidaemia, family history of CHD, smoking and metabolite batch. The arrows ↑/↓, respectively, indicate an increase or a decrease of the metabolite levels in the plasma of CHD patients compared to those of control subjects.
P-value < 0.05 was considered statistically significant.
